# Reducing the Time Requirement of k-Means Algorithm

**DOI:** 10.1371/journal.pone.0049946

**Published:** 2012-12-11

**Authors:** Victor Chukwudi Osamor, Ezekiel Femi Adebiyi, Jelilli Olarenwaju Oyelade, Seydou Doumbia

**Affiliations:** 1 Department of Computer and Information Sciences, College of Science and Technology, Covenant University, Ota, Ogun State, Nigeria; 2 Malaria Research and Training Center (MRTC), University of Bamako, Mali; Université de Nantes, France

## Abstract

Traditional k-means and most k-means variants are still computationally expensive for large datasets, such as microarray data, which have large datasets with large dimension size *d*. In k-means clustering, we are given a set of *n* data points in *d*-dimensional space R*^d^* and an integer k. The problem is to determine a set of k points in R*^d^*, called centers, so as to minimize the mean squared distance from each data point to its nearest center. In this work, we develop a novel k-means algorithm, which is simple but more efficient than the traditional k-means and the recent enhanced k-means. Our new algorithm is based on the recently established relationship between principal component analysis and the k-means clustering. We provided the correctness proof for this algorithm. Results obtained from testing the algorithm on three biological data and six non-biological data (three of these data are real, while the other three are simulated) also indicate that our algorithm is empirically faster than other known k-means algorithms. We assessed the quality of our algorithm clusters against the clusters of a known structure using the Hubert-Arabie Adjusted Rand index (ARI_HA_). We found that when k is close to d, the quality is good (ARI_HA_>0.8) and when k is not close to d, the quality of our new k-means algorithm is excellent (ARI_HA_>0.9). In this paper, emphases are on the reduction of the time requirement of the k-means algorithm and its application to microarray data due to the desire to create a tool for clustering and malaria research. However, the new clustering algorithm can be used for other clustering needs as long as an appropriate measure of distance between the centroids and the members is used. This has been demonstrated in this work on six non-biological data.

## Introduction

Clustering is the unsupervised grouping of objects into classes without any *a priori* knowledge of the datasets to be analyzed. A clustering algorithm is either hierarchical or partitional. Hierarchical algorithms create successive clusters using previously established clusters, whereas partitional algorithms determine all clusters at once. For the hierarchical variants, we have the agglomerative and divisive clustering. However, in partitional clustering, we have QT (Quality Threshold) clustering [1], Self Organising Map (SOM) [2] and Standard k-means [3], [4], which have been evolving in recent years for high dimensional data analysis [5], [6], [7], [8], [9], [10], [11]. An overview on clustering algorithms for expression data can be found in Yona *et al.* [12]. Examples of variants of k-means algorithms that attempted to enhance the traditional k-means algorithm via improved initial centre can be found in Deelers and Auwatanamongkol [13], Nazeer and Sabastian [14] and Yedla *et al* [15]. Lastly, Kumar *et al.* [16] in a recent work, enhanced the k-means clustering algorithm using red black tree and min-heap.

The traditional k-means algorithm requires in expectation *O(nkl)* run time where *l* is the number of k-means iterations. This time was said to be reduced by Fahim *et al.* [17] to *O(nk)*. Fahim *et al.*, used *n* 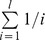 to estimate the total number of data points for each iteration that changed their clusters during the number of k-means iterations, *l*, thereby deducing that the cost of using their enhanced k-means algorithms is approximately *O(nk)*. The k-means algorithm described in Nazeer and Sebastian [14] also runs in *O(nk)* while the one in Yedla *et al.* [15] runs in *O(nlogn)*.

For efficient and effective analysis of microarray data, we developed a novel Pearson correlation-based Metric Matrices k-means (MMk-means). We showed that the algorithm is correct. Experimental results show that it has a better run-time than the Traditional k-means and other variants of k-means algorithm like Overlapped and Enhanced k-means algorithms developed in [17]. Furthermore, the new clustering algorithm can be used for other clustering needs as long as an appropriate measure of distance between the centroids and the members is used. This has been demonstrated in this work on six non-biological data.

## Methods

### Notation


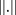
 denotes the Euclidean norm of a vector. The trace of a matrix *X, i.e*., the sum of its diagonal elements, is denoted as trace (A). The Frobenius norm of a matrix 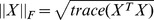. I_n_ denotes identity matrix of order n.

### Basic Definitions

#### Metric Matrix (MM)

A kxk matrix encapsulates the correlation coefficient (r) between the centroids of the previous (pm_j_) and current (m_j_) iterations respectively, where 0<j≤k.

#### Ding-He Interval

It is an interval, obtained by Ding and He [18], [19], used in our new k-means algorithm to determine when a cluster must remain without further clustering or be subjected to further clustering.

#### MMk-means Iterations (MMI)

The number of k-means iterations required before the Ding-He interval is applied in our new k-means algorithm.

#### diff_j_

An absolute value obtained from the subtraction of the current iteration eigenvalues (e_j_) from the previous iteration eigenvalues (pe_j_) which serves as an indicator to terminate clustering for each cluster. Each eigenvalues set is obtained from the corresponding Metric Matrix.

### Some Set Notations

set[j]: 1≤j≤k is the set referring to cluster *j*.

add[*i*]: Is a function to add data point into a cluster, where *i* is the index of the data.

set[j].n*_j_*: Is the size of cluster *j*, that is, number of data points in a cluster *j*.

### Algorithm Design

Our MMk-means algorithm runs like the Traditional k-means algorithm except that it is equipped with a mechanism to determine when a cluster is stable, that is, its membership data points will always remain in the same cluster in each subsequent iteration. This is an improvement over the Overlapped and Enhanced variants of k-means algorithms introduced by Fahim *et al.* [17]. They equipped their algorithms with the ability to detect the stability of a data point but MMk-means is equipped with the mechanism to detect the stability of a cluster representing a whole bunch of data points.

We use the recently established relationship between principal component analysis and k-means clustering to design a mechanism for determining when the whole data points in a cluster are stable. We create a covariance matrix (MM of r's), a result of computing the Pearson product moment correlation coefficient between the k centroids of the previous and current iterations and then deduce k previous and current iterations eigenvalues. The difference of these eigenvalues for each cluster is computed and checked to see if it satisfies (that is, lies within) the Ding-He interval [18], [19]. If it does, the corresponding cluster is considered stable and there is no need to compute its data point distances with the current centroid of the cluster or the rest k-1 centroids.

**Figure 1:**
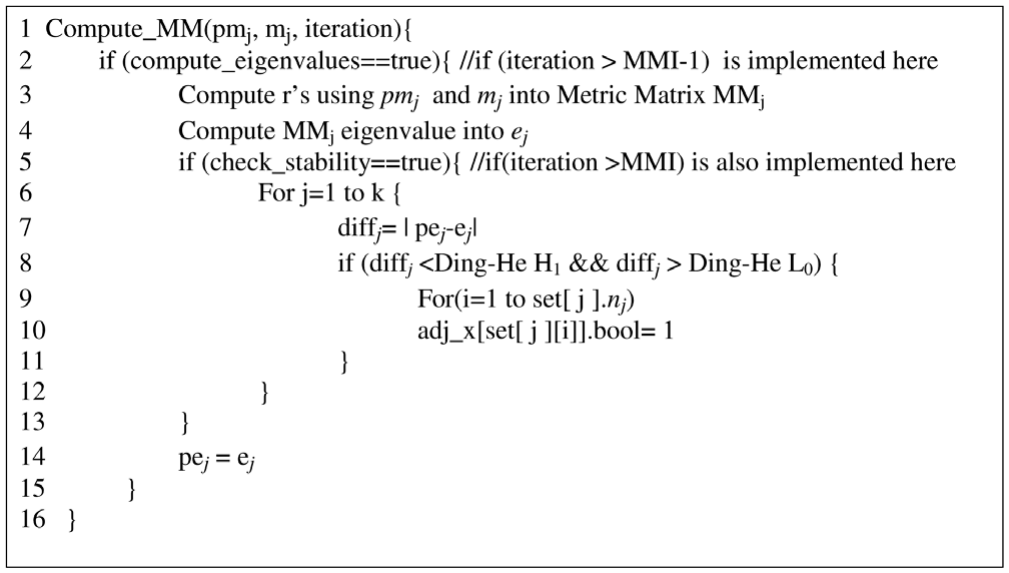
Pseudocode of our Compute_MM Sub-program for *MMk-means*. We create a covariance matrix, computing the Pearson product moment correlation coefficient between the k centroids of the previous and current iterations and then deduce k previous and current iterations eigenvalues. The difference of these eigenvalues for each cluster is computed and checked to see if it satisfies the *Ding-H*e interval.

The mechanism explained above is prescribed in the subprocedure Compute_MM of *Figure* 1 and this function is being executed when the current total iterations number is greater than

MMI-1. For any *n* dataset points, given the total number of k-means iteration *l* required, we can actually set MMI = *l*/2, but note that *l* is unknown, until a traditional k-means algorithm is executed. We know that for a given clustering procedure, k-means algorithm aims to minimize the first Mean Squared Error (MSE_1_), through a number of iterations, *l*, distributing all data points into clusters, to arrive at an optimal (minimized) Mean Squared Error (*MSE_l_*). Therefore, we estimate the required MMk-means Iteration (*MMI*) to be bounded by 0<*MMI*≤MSE_1_.*k*/MSE*_l_*. Note that for a given set of *n* data points, we can form the *n*-by-*d* matrix *X* = [x_1_,…, x_n_] and the first iteration of a traditional k-means algorithm can be used to determine MSE_1_ in *O(nk)* time. Ding and He [18], [19] provided tightly upper and lower bounds for the optimal MSE*_l_*. From these, we can compute MSE*_l_* in *O(n)* time. Empirical testing followed by personal communication with Ding, C. shows that the deduced technique does not hold for large k and data with high dimensional *d*. So we still cannot estimate MSE*_l_* for all k and *d* as we desire in our new k-means algorithm.

In a previous work, that led to the results in [18], [19], Zha et al. [20] obtained that:

#### Theorem 1

*The optimal Mean Squared Error (MSE_l_) is bounded from below by*

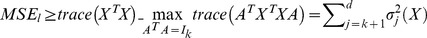
(1)
*where* 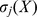 *is the j largest singular value of X and A is an arbitrary orthonormal matrix.*

Ding and He [18], [19] indicated that the lower bound in theorem 1 is not asymptotically tight. They however provided (derivatively) empirical evidence in pages 35 and 500 respectively that as the number of cluster increases (that is *k*), the lower bound in theorem 1 becomes less tight, but still around 1.5% of the optimal values. This can also be shown when the dimension of the data increases and when both increase, but they do not provide logical argument to prove this. We provide this proof in the following observation.

An important result, that we shall see soon, how it relates centroid of each partition 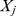 to an eigenvalue, is given by Fan [21] and stated below:

#### Theorem 2

*Let H be a symmetric matrix with eigenvalues,* 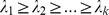 *and the corresponding eigenvector* U = [u_1_,…,u_d_]. *Then*

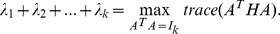

Moreover, the optimal A^*^ is given by 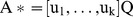 *with Q an arbitrary orthogonal matrix.*

#### Observation 1

*From theorem 1 above, we observed that although the equation does not correspondingly estimate MSE_l_ for large k and high d, it possesses a better analytical distribution that mimic the series needed to estimate MSE_l_ for large k and high d.*

*Proof*. From theorem 1, we know that
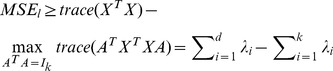
(2)using theorem 2 above and the fact that the sum of the eigenvalues of any (square) matrix is equal to the trace of the matrix. We can see from equation (2) that since 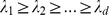 from theorem 2, for large k (though still ≪d), very few largest eigenvalues are the ones subtracted. For larger k and higher d, the estimation in equation (2) is kept balance by the addition of eigenvalues at the tail and the subtraction of very few (in normal practical setting) at the rear of the series. This way, the lower bound in theorem 1 though may be less tight is still within the excellent reach of the optimal value. 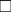


Using this observation, in the following, we are able to estimate more accurately a multiplier, we called *m*, that is useful in the prediction of MSE*_l_* from MSE_1_ and consequently determine *MMI*.

#### Observation 2

*From* *equation (1)* *, we can estimate*

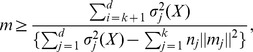

**Figure 2:**
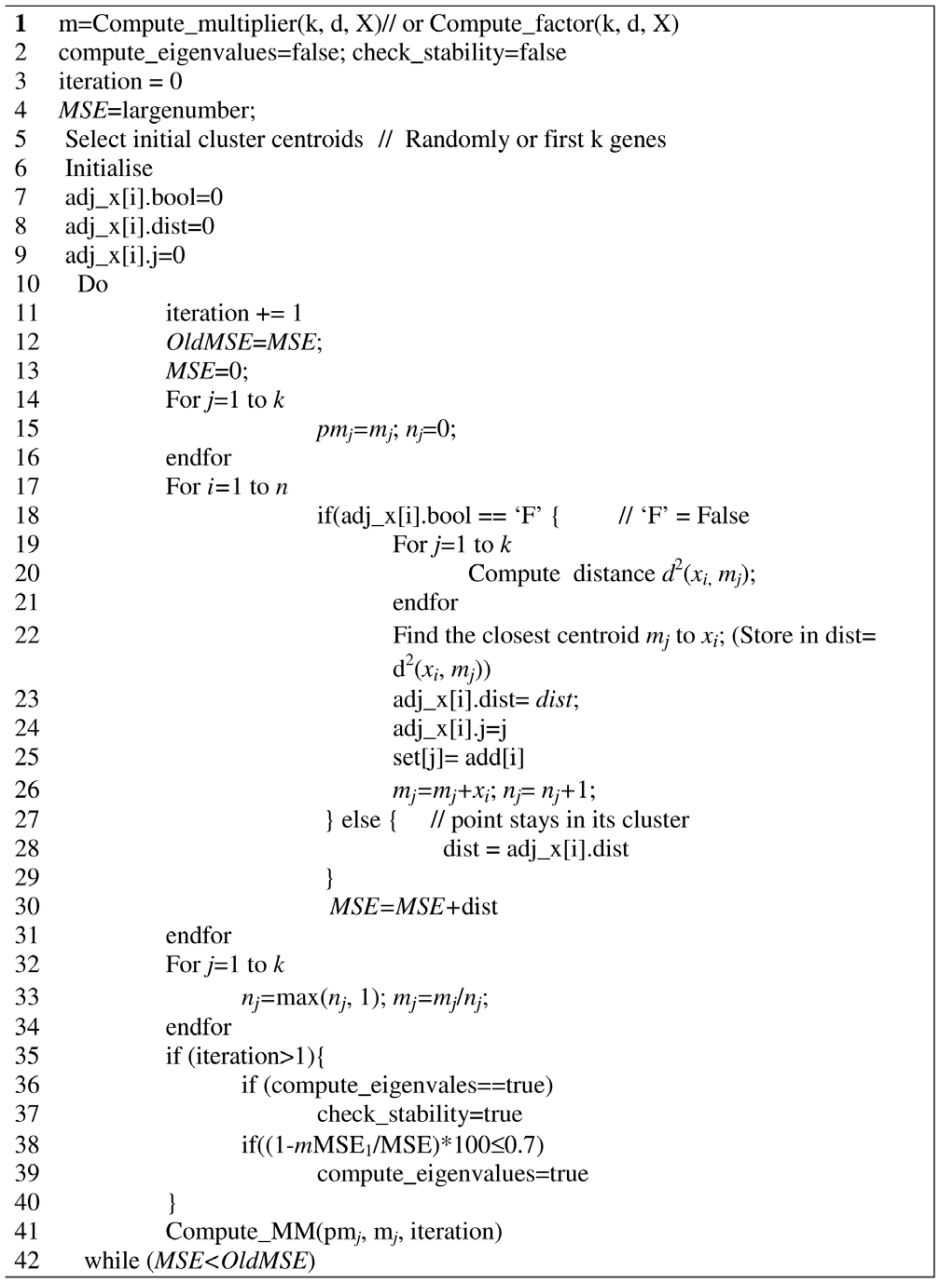
Pseudocode of our main program for *MMk-means*. It runs similar to the traditional k-means except that it is equipped with a metric matrices based mechanism to determine when a cluster is stable (that is, its members will not move from this cluster in subsequent iteration). This mechanism is implemented in sub-procedure Compute_MM of *Figure* 1. We use the theory developed by Zha *et al.* [20] from the singular values of the matrix X of the input data points to determine when it is appropriate to execute Compute_MM during the k-means iterations. This is implemented in lines 34–40.

*where* 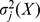 *is the j largest singular value of x, n_j_ is the size of the data vectors in cluster j and m_j_ is the mean vector of these data vectors. And consequently find MSE_l_ ∼ mMSE*_1_. *We encapsulate the computation of the multiplier m in an implicit subprocedure Compute_multiplier in line 1 of* *Figure 2* *.*

#### Proof

Recalling what we stated earlier on in the body of the paper, it is known that for a given clustering procedure, k-means algorithm aims to minimize the first Mean Squared Error (MSE_1_), through a number of iterations, *l*, distributing all data points into clusters, to arrive at an optimal (minimized) Mean Squared Error (*MSE_l_*).

Zha *et al.* [20] in their attempt to prove theorem 1 (stated in the paper main body) showed that theoretically
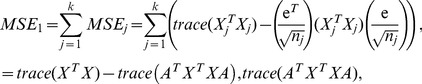
(4)where A is an n x k orthonormal matrix given by
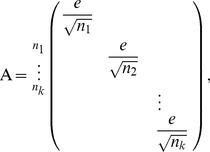
(5)e is a vector of appropriate dimension with all elements equal to one and 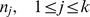 are number of data points in each cluster. They also showed that 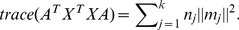
(6)So *MSE_1_* in equation (4) becomes
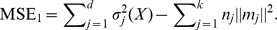
Minimizing equation (4) and using theorem 2 and equation (6) above, Zha *et al.* [20] showed that 
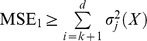
 as given in theorem 1 (of the main body of this paper).

So we can estimate the factor *m* that relates *MSE_l_* and MSE_l_ as follows:
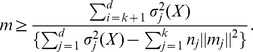
And therefore from observation 1, for large *k* and *d*, we can estimate *MSE_l_* from MSE_1_ and consequently have 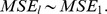 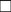


#### Observation 3

*For each iteration, given* 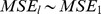 *, we can determine MMI, by estimating the distance of the current iteration MSE away from the final and optimal MSE,* 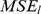 *and the first instance when* 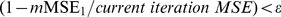 *, where* 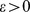 *, MMI is equal to the current total iterations number. This is also implemented in lines 35–40 of* *Figure 2* *.*

#### Proof

The most widely used convergence criteria for the k-means algorithm is minimizing the *MSE*. Selim and Ismail [22] provided a rigorous proof of the finite convergence of the k-means type algorithm for any metric. We know that every convergent sequence (with limit s, say) is a Cauchy sequence, since, given any real number 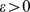, beyond some fixed point, every term of sequence is within distance 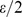
of s, so any two terms of the sequence are within distance ε of each other. By definition, a Cauchy sequence is a sequence, 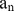 such that for any 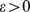
there exists an integer K≥1 such that 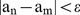 for all n and m with 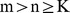.

MMI that we seek in this observation is K, since 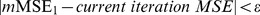 following the Cauchy sequence definition. This can be rewritten as 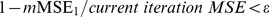. This completes the proof. 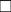


Using the devices enumerated above, our new k-means algorithm is presented in *Figures* 1 *and* *2*. We now prove the correctness of this algorithm.

### MMk-means algorithm correctness proof

To prove the correctness of our new and novel k-means algorithm, we will need the following definitions from Kumar *et al.* [23] and Kanungo et al. [24].

#### Definition 1

*Given a set of k points K, which we also denote as centers, define the k-means cost of X, set of n points in d-dimensional space* 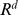 *, with respect to K,* 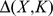 *as*

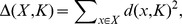

*where d(x, K) denotes the distance between x and the closest point to x in K.*

#### Definition 2

*For a set of points X, define the centroid, C(X), of X as the point*


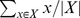
 *. For any point* 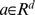 *, it follows that* 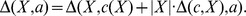


Let the centroids at each k-means iteration be 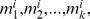 1≤i≤*l* where 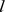 is the total number of k-means iterations. Now, we will also need the following lemma. The following lemma shows the mathematical correctness of our key sub program, Compute_MM of Figure 1, which encapsulates the mechanism we used to identify stable partitions in our new MMk-means algorithm.

#### Lemma 1

*For a partition* 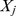 *at* 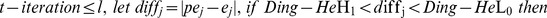 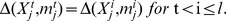


*Proof*. Note that the Metric Matrix, *MM* in sub-procedure Compute_MM of *Figure* 1, which is the key mechanism we used to identify stable partitions, is the k x k correlation coefficient matrix generated between the centroids of the previous and current iterations of the k-means algorithm. Note further that this matrix is a covariance matrix [25].

Note that the minimization of equation (4) is equivalent to
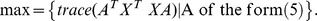

Equations (4) and (6) relate minimized MSE to maximizing the sum of the centriods. Note also that equation (6) and theorem 2 above relate centriods of each partition to an eigenvalue. Iteratively, *MM* in Compute_MM relates centriods of previous and current iterations respectively and therefore from equation (6) and theorem 2, its eigenvalues characterize the iterative minimized MSE of each partition and 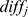 is an estimate of how close is the minimized MSE for a partition (in term of its centroid) to the optimal one. Since Ding and He [18], [19] had shown an upper and lower bound to expect this, then if 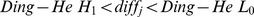, the centroid of the corresponding partition 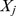 virtually does not change in subsequent iterations.

This translates to 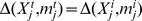 for 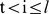 from definition 2. 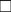


The following is now the correctness proof for the MMk-means algorithm.

#### Theorem 3

*Given a point set X, MMk-means returns a k-means solution on input X.*

*Proof*. We should note that our algorithm maintains the following loop invariant:

**Invariant:** Let
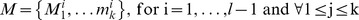

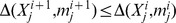
,
2. **The set X is a subset of** 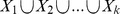.

It is straight forward to note that for 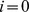, the invariant holds. Now, let assume that the invariant holds for some fixed 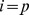, its remains to show that the invariant holds for 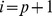 as well, then we are done.

Based on our assumption, for 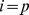,
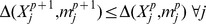
(7)For 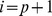, we have to show for every *j* that it is either
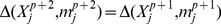
(8)or
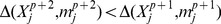
(9)Note that for a partition 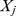, if 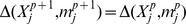 from (1) above then using lemma 1, 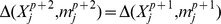 for all iteration later on. This proves (8) above.

Now if 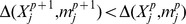 from (7), it remains to prove that

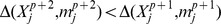
 or

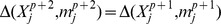

Lemma 1 indicates the condition to expect ii), so we are done as regards this. If this condition is not valid for a particular partition *X_j_* then 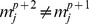. From definition 1,
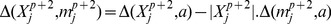
(10)and
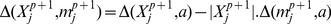
(11)for any point 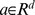. Note that for our MMk-means algorithm and infact any other k-means algorithm, if 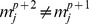, then 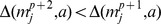
and therefore from equations (10) and (11), 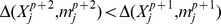. This completes the proof for (9) above.

It now remains to show that for each iteration *i* in our MMk-means algorithm, the input set *X* is a subset of 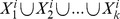. This is actually straight forward. Note that for an iteration *i*, 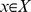, there exist only one closest 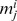 centriod to *x* from *M* for a particular partition 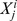, such that 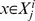. This shows that all 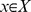 belongs to a partition 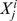 for 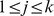 and therefore *X* is a subset of 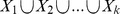. 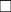


## Results and Discussion

Using C++, we implemented the three variants of k-means algorithms, namely, the Traditional, Overlapped and Enhanced k-means following Fahim et al. [17] design. We also implemented the fourth one, our MMk-means algorithm using C++ and MATLAB. See the additional file S1 of this paper for the source codes of these programs. Ding and He [18], [19] experimentally determined an interval: 0.5–1.5%, which indicates when a cluster is optimally equal to the expected ones. We used this in our Compute_MM of *Figure* 1, where we set L_0_ = 0.5% and H_1_ = 1.5%. Finally, *Observation 3* is implemented with experimentally determined ε = 0.007.

We tested the algorithms using normalized microarray expression data at varying timepoints for *P. falciparum* microarray experiment data from [26] and [27] as depicted in *Table* 1. See additional file S2 for a zip file containing all the microarray data. The number of genes ranges from 4313–5159 while the number of time-points is from 16–53. The values of k include 15, 17, 19, 20, 21, 23, 25. The system used is a DeLL computer, INTEL® CORE™ DUO CPU T2300 @1.66 GHz, 512 RAM, 80 GB HDD.

**Table 1:** Short statistics on the three microarray experimental data used in the testing of our algorithm and the other three variants of k-means algorithm.

P.f Microarray Experimental data	Total No Of Genes	Time points
Bozdech et al, (2003)- 3D7 strain data	4596	53
Bozdech et al., (2003) – Hb3 strain data	4313	48
Le Roch et al, (2003) 3D7 strain data	5159	16

The second and third columns indicate the total number of genes covered in each experiment and the number of points (at equal interval) at which the genes transcriptional expression are measured.

**Figure 3:**
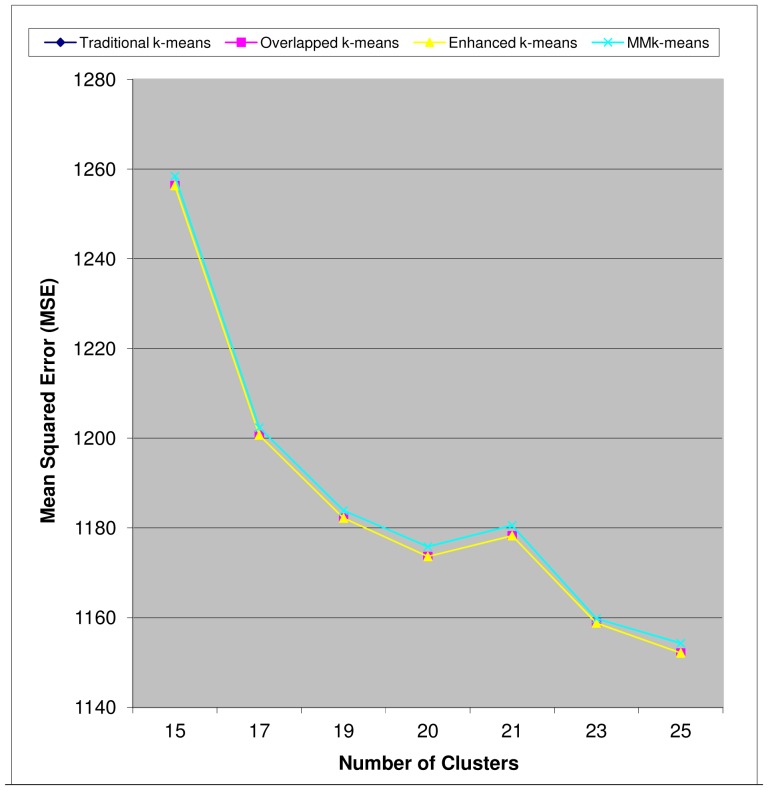
Quality of Clusters (Bozdech *et al.*, *P.f* 3D7 Microarray Dataset). The qualities of clusters for the four algorithms are similar. The MSE decreases gradually as the number of clusters increases except for k = 21 that has a higher MSE than when k = 20.

The plots of minimized Mean Standard Error (MSE) versus k values help to measure clusters quality (that is, its effectiveness) and run time (in sec) versus k help to measure each algorithm's efficiency empirically. For the malaria microarray data from Bozdech *et al.* [26], these plots are shown in *Figures* 3 *and* 4. These results are similar to what was obtained from the other malaria microarray data as indicated in *Table* 1. It was observed that eigenvalues of the Metric Matrix, *MM* decrease along the diagonal matrix from top to bottom for all iterations except the last one and change interestingly at the last iteration by increasing from top to bottom. It should be noted that the stability condition for cluster as measured by *diff_j_* of line 7 in *Figure* 1 does not apply appropriately to negative gene expression values as we have in Le Roch *et al.* [27] data. The theoretical reason is given in [18], [19]. We observed that, nevertheless, our new algorithm compared excellently to the Traditional k-means.

**Figure 4:**
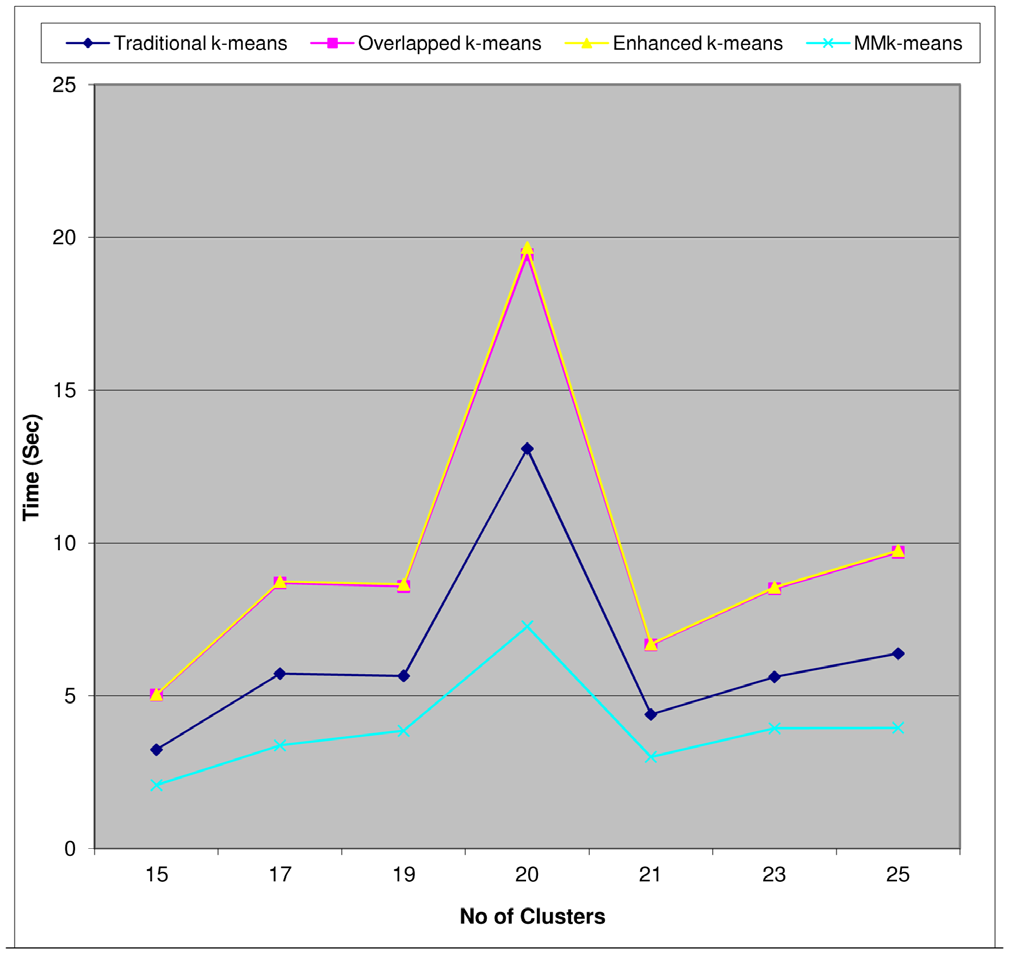
Execution Time (Bozdech *et al.*, *P.f* 3D7 Microarray Dataset). The plot shows that our MMk-means has the fastest run-time for tested number of clusters, 15≤k≤25. Comparatively, k = 20 took the longest run-time for all the four algorithms, implying that this is a function of the nature of the data under consideration.

We found out that the algorithms of Fahim *et al.* [17] were slower than the Traditional k-means contrary to the claim of the authors. This observation made us to take a critical look at the design of the two algorithms of Fahim et al. [17] theoretically. Fahim *et al.* designed two new variants of k-means algorithms noting that if the distance between a data point and the current centroid (new center) of the cluster that it was assigned to in the previous iteration is less than or equal to the distance of the data point to its previous centroid (old centre), then the point remains in that cluster and there is no need to compute its distance to the other *k*-1 centers. To do this, they introduced two arrays, namely *Clusterid* and *Pointdis* to keep track of the centroid to which each point is assigned to and the distance between this point and its centroid. Fahim *et al.* [17], used *n* 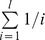 to estimate the total number of data points for each iteration that moved from their clusters during the number of k-means iterations, *l* and showed that the cost of using an enhanced k-means algorithm is approximately *O(nk)*. We observed that the total number of data points for each iteration that moved from their clusters during the k-means iterations is not strictly monotonically decreasing and thus their Overlapped and Enhanced k-means algorithm eventually still costs *O(nkl)* run time in expectation. From the foregone, the two algorithms have the same asymptotic run time as the Traditional k-means algorithm but in practise (from our experimental experience) slower than the traditional one.

Whenever k (number of clusters) <*d* (dimension or timepoints), effective clustering is achieved for the four algorithms and our MMk-means has the best empirical runtime. Overlapped and Enhanced k-means are the slowest in all cases. Empty clusters are created by all the algorithms if k>*d* as the clustering becomes irregular, similar to results for 15>k>25 by Le Roch *et al.* [27].

To further ascertain the quality of our new algorithm on the three microarray data of *Table 1*, we assessed the quality of its clusters and that of Enhanced and Overlapped k-means respectively, against the clusters from the Traditional k-means, using the Hubert-Arabie Adjusted Rand index (ARI_HA_) [28]. The result of this assessment is given in *Table* 2 for biological data and in *Table* 3 for non-biological data. Considering each biological data, Bozdech et al. 3D7 and HB3 strains [26] and Le Roch et al. [27], we used two values of k to demonstrate the effect of changing k values on the clusters quality of the clustering algorithms. In a separate column, we also compare the structure of the Enhanced k-means with that of Overlapped k-means. We found out that Enhanced and Overlapped k-means respectively produced similar clusters and their structures are similar to that of the Traditional k-means. For MMk-means, this is also the case and we found categorically that when k is close to d, the quality of its clusters is good (ARI_HA_>0.8) and when k is not close to d, the quality is excellent (ARI_HA_>0.9).

**Table 2:** Hubert-Arabie Adjusted Rand Index (ARI_HA_) Cluster Quality Computation Result for Biological and Non-biological data.

	Traditional k-means						Enhanced k-means					
	Bozdech et al.(2003)-3D7 strain		Bozdech et al.(2003)-HB3 strain		Le Roch et al. (2003)		Bozdech et al.(2003)-3D7 strain		Bozdech et al.(2003)-HB3 strain		Le Roch et al. (2003)	
	K = 15	k = 20	k = 15	k = 20	k = 10	k = 15	k = 15	k = 20	k = 15	k = 20	k = 10	k = 15
**MMk-means**	0.9480	0.9170	0.9068	0.6488	0.9352	0.6643						
**Enhanced** **k-means**	0.9935	1.0000	0.9901	0.9967	0.9717	0.9728						
**Overlapped** **k-means**	0.9635	1.0000	0.9707	0.9920	0.8837	0.8682	0.9636	1.0000	0.9720	0.9917	0.8891	0.8916

For each data, Bozdech et al. 3D7 and HB3 strains [26] and Le Roch et al. [27], we used two values of k to demonstrate the effect of changing k values on the clusters quality of the clustering algorithms. We considered the structure of the Traditional k-means as the known structure and compare the clusters of MM, Enhanced and Overlapped k-means respectively with it. In a separate (last) column, we also compare the structure of the Enhanced k-means with that of Overlapped k-means.

**Table 3:** Hubert-Arabie Adjusted Rand Index (ARI_HA_) Cluster Quality Computation Result for Non-biological data.

	Traditional k-means						Enhanced k-means					
	Abalone		Wind		Letter		Abalone		Wind		Letter	
	K = 5	k = 7	k = 5	k = 12	k = 5	k = 10	k = 5	k = 7	k = 5	k = 12	k = 5	k = 10
**MMk-means**	0.8472	0.6045	1.0000	0.9205	0.8623	0.8015						
**Enhanced** **k-means**	0.9454	0.9837	0.9992	0.9997	0.9930	1.0000						
**Overlapped** **k-means**	0.9540	0.9004	0.9895	0.9821	0.9875	1.0000	0.9544	0.9064	0.9904	0.9818	0.9879	1.0000

For each data, Abalone, Wind and Letter as described in Table 4 below, we used two values of k to demonstrate the effect of changing k values on the clusters quality of the clustering algorithms. We considered the structure of the Traditional k-means as the known structure and compare the clusters of MM, Enhanced and Overlapped k-means respectively with it. In a separate (last) column, we also compare the structure of the Enhanced k-means with that of Overlapped k-means.

In Osamor *et al.* [29], we demonstrated the biological characteristics of our new algorithm against other well known k-means clustering algorithms. Interestingly, from this application, we discovered a new functional group for some set of genes of *P. falciparum*.

To test the behavior of our new algorithm on non-biological data, we used first, the data of Fahim et al. [17]. Details on these datasets are given in *Table* 4. The result of this exercise is given in *Table* 3. The quality of MMk-means clusters is similar to what we observed from that of the biological data. Next, we tested MMk-means algorithm on three large simulated datasets of 50 dimensional size and with 10000, 30000, 50000 items respectively. The result is shown in *Table* 5. MMk-means is empirically efficient than all other three algorithms and the quality of MMk-means clusters is again similar to what we observed from that of the biological data.

**Table 4:** Non-Biological data used for testing our algorithm and the other three variants of k-means algorithm.

Dataset	No of Records	No of Attributes
Abalone	4177	7
Wind	6574	12
Letter	20000	16

Abalone dataset described with 8 attributes represents physical measurements of abalone (sea organism). Wind dataset described by 12 attributes represents measurements on wind from 1/1/1961 to 31/12/1978. Letter dataset represents the image of English capital letters described by 16 primitive numerical attributes (statistical moments and edge counts).

**Table 5:** Performance comparison for all types of k-means algorithms considered for very large data sets.

Input Size of Data	Run time (in sec) versus k				
4.3 MB (10,000×50)	k	Traditional_k-means	MMk-means	Overlapped k-means	Enhanced k-means
10	57.252	54.226	87.875	85.738	
20	69.498	59.647	87.875	106.439	
30	85.910	82.291	24.769	128.873	
40	72.603	69.324	109.653	111.993	
12.9 MB (30,000×50)	k	Traditional_k-means	MMk-means	Overlapped k-means	Enhanced k-means
10	120.167	115.152	191.366	184.580	
20	215.780	209.612	319.786	355.417	
30	404.152	396.536	592.648	611.384	
40	297.307	286.023	428.004	424.759	
21.5 MB (50,000×50)	k	Traditional_k-means	MMk-means	Overlapped k-means	Enhanced k-means
10	250.069	242.406	378.661	385.697	
20	520.091	484.117	696.014	704.657	
30	550.652	539.308	816.478	853.684	
40	641.117	631.755	971.559	961.075	

This constitute simulation of three large data sets in the order of; 10,000×50, 30,000×50 and 50,000×50 dimension. The range of K used is 10≤K≤40 for the four algorithms.

### Conclusion

To achieve efficient but also effective analysis of microarray data, we developed a novel Pearson correlation-based Metric Matrices k-means (MMk-means). We provided the correctness proof of this algorithm. Experimental results show that it has a better run-time than the Traditional k-means and other variants of k-means algorithm like Overlapped and Enhanced k-means algorithms developed in [17].

It must be pointed out that the results (extended theories and experimental) of this work provide additional toolkits to analyze successfully high dimensional datasets, which of recent, are of incredible growth [10], [11], [30]. However, the new clustering algorithm can be used for other clustering needs as long as an appropriate measure of distance between the centroids and the members is used. This has been demonstrated in this work on three moderate size and three heavy non-biological data.

## Supporting Information

File S1 **Traditional, Overlapped, Enhanced and MM- kmeans algorithms C++ codes.** Kmeansprograms.zip contains Traditionalkmeans.cpp, Overlappedkmeans.cpp, Enhancedkmeans.cpp and Mmkmeansmmi.cpp. These programs are implemented using Borland C++ version 5.0 and MATLAB version 7.0. The steps on how to run the programs are stated at the beginning of the program files in the zip.
(ZIP)

File S2 **The three microarray experimental data used in the testing of our algorithm and the other three variants of k-means algorithm.** The files in this Data.zip are as follows. These files can best be viewed using MS Excel or Notepad. 1. Bozdech3D7.txt from Bozdech et al., (2003) for *P.falciparum* 3D7 strain microarray data 2. BozdechHB3.txt from Bozdech et al., (2003) for *P.falciparum* HB3 strain microarray data 3. Leroch3D7.txt from Le Roch et al., (2003) for *P.falciparum* 3D7 strain microarray data.
(ZIP)
